# Association of *nNOS* Gene Polymorphism with Ischemic Stroke in Han Chinese of North China

**DOI:** 10.1155/2013/891581

**Published:** 2013-09-03

**Authors:** Yingjie Dai, Zhiyi He, Rubo Sui, Zhilin Jiang, Shanshan Ma

**Affiliations:** ^1^Department of Neurology, The First Affiliated Hospital of China Medical University, Shenyang 110001, China; ^2^Department of Neurology, NO 202 Hospital of People's Liberation Army of China, Shenyang 110001, China; ^3^Department of Neurology, Jinqiu Hospital of China Liaoning Province, Shenyang 110001, China

## Abstract

Nitric oxide (NO) is an important messenger molecule and effector molecule. This study aimed to investigate the relation of neuronal nitric oxide synthase (nNOS) gene polymorphism with ischemic stroke in Han Chinese of North China. This was a case-control study. A total of 413 patients with ischemic stroke were recruited from Han Chinese of North China. There were 201 males and 212 females. In addition, 477 healthy subjects served as controls including 224 males and 253 females. Multiplex SNaPshot was employed to detect *nNOS* gene polymorphism (rs2293050, rs2139733, rs7308402, and rs1483757). Results showed that the rs1483757, rs2139733, and rs2293050 genotypes and allele frequencies were comparable between patients and controls. However, ischemic stroke patients had significantly reduced AG genotype and A allele frequency when compared with controls (*P* = 0.037, *P* = 0.041). After adjusting confounding factors (gender, age, smoking, history of drinking, hypertension, and diabetes), AG genotype and A allele were still related to ischemic stroke (OR = 0.572, 95% CI: 0.335–0.978, *P* = 0.041; OR = 0.611, 95% C: 0.378–0.985, and *P* = 0.041) and both were found to be protective factors. Our results showed that rs7308402 gene polymorphism of nNOS is related to ischemic stroke in Han Chinese of North China.

## 1. Introduction

Stroke has a high morbidity, high mortality, high disability, and high recurrence rate and has been a major cause of disability and death worldwide [1]. In China, there are 1500000 to 2000000 new stroke cases every year and 70% of these are ischemic stroke (IS) cases [2]. It is well known that atherosclerosis, small vessel disease, and arrhythmia are common causes of IS [3], and hypertension, diabetes, and smoking have been found to be risk factors of IS [4]. Increasing evidence shows that IS is caused by multiple factors and as a result of interaction between genetic and environmental factors. It has been confirmed that mutations of *MTHFR* gene [5], *ApoE* gene [6], *PDE4D* gene [7], and *ALOX5AP* [8] are related to the pathogenesis of IS.

NO is an important messenger molecule and effector molecule. In organisms, NO may serve as a messenger, mediator, or cellular modulator and possesses extensive biological activities [9]. The biosynthesis of NO is regulated by nitric oxide synthase (NOS). According to the biological characteristics and encoding genes, NOS can be classified into neural NOS (nNOS, *NOS1*), inducible NOS (iNOS, NOS2), and endothelial (NOS, NOS3). eNOS is mainly expressed in the endothelial cells. Under the physiological condition, eNOS derived NO is required for maintenance of circulation system. It can dilate blood vessels, inhibit platelet aggregation, and suppress proliferation of vascular smooth muscle cells exerting antihypertensive and anti-inflammatory effects. Thus, *eNOS* gene has been regarded as a candidate gene of IS. It has been found that eNOS4a and *G894T* mutations are the genetic markers of IS [10]. In recent years, animal studies reveal that nNOS derived NO plays a vasoprotective role in the atherosclerosis [11–14]. nNOS derived NO in nonadrenal, noncholinergic autonomic nerves may activate systemic vasculature and together with eNOS derived NO, dilate peripheral, and cerebral blood vessel [9, 15, 16] and is also involved in the control of blood pressure [17, 18]. In the cerebral infarction model, *NOS1*-deficient mice had smaller infarct ratio and milder neurological dysfunction when compared with controls, suggesting that *NOS1* is detrimental for cerebral infarction [19, 20]. Thus, nNOS may influence the susceptibility to stroke in a mechanism complementary but different from that of eNOS.

The human *NOS1* gene was first identified in the neurons of the brain and thus is also known as neural NOS (nNOS) [21]. It has been confirmed that nNOS is expressed in not only the brain and peripheral nitrogen source nerves but also the kidney, heart, muscular skeletal muscles, vascular smooth muscle cells, and endothelial cells. Human *NOS1* (nNOS) gene is mapped to 12q24.2, and 240 kb in length. It contains 29 exons and 28 introns. To date, few studies have been conducted to investigate the relationship between *nNOS* gene polymorphism and pathogenesis of IS. Recently, Manso et al. [22] investigated the *NOS1* gene polymorphism in a Portuguese population. Their results showed that the *NOS1* gene polymorphism (rs1483757, rs7308402, rs2293050, and rs2139733) was closely related to the pathogenesis of IS. Thus, it is necessary to confirm this finding in different populations. To date, no study has been carried out to explore the relation of *NOS* gene polymorphism with the pathogenesis of IS in Chinese. This case-control study was undertaken to investigate the association between *NOS1* gene polymorphism and IS in Han Chinese of North China. Furthermore, stratification analysis was done according to the gender. Our findings may provide evidence for the prevention of stroke.

## 2. Materials and Methods

### 2.1. Subjects

Acute ischemic stroke patients (*n* = 413) were recruited from the Department of Neurology of First Affiliated Hospital of China Medical University and NO 202 Hospital of People's Liberation Army of China. There were 201 males and 212 females. Focal neurological deficits were abrupt and acute and continued for more than 24 h. Ischemic stroke was confirmed by brain MRI and/or cranial CT scan. Transient ischemic attack, cerebral embolism, hemorrhagic infarction, cerebral hemorrhage, and subarachnoid hemorrhage were excluded. In addition, cerebral infarction due to cardiogenic events, arteritis, tumors, drugs, trauma, vascular malformations, hematological diseases, or aneurysm was excluded. Concomitant liver and kidney diseases and thyroid disease were not found in these patients. Diagnosis was done by neurologists. Controls (*n* = 477) were the healthy subjects who received routine physical examination in the First Affiliated Hospital of China Medical University. There were 224 males and 253 females. Controls and patients were matched in age and gender. History reviewing, physical examination, and clinical examinations were done in these controls and cerebrovascular diseases; other neurological diseases; kidney/liver diseases, hematological diseases, tumors, peripheral vascular diseases, and autoimmune diseases were excluded from these controls. There was no kinship among all these subjects who were Han Chinese in Liaoning Province, a region of North China. This study was approved by the Ethic Committee of China Medical University and 202 Hospital, and informed consent was obtained from all subjects before study. Questionnaire, physical examination, and laboratory examinations were performed to acquire clinical information of these subjects including age, gender, height, body weight, blood pressure, blood lipid, fasting blood glucose, past history, and history of smoking and drinking (Table 1).

### 2.2. Genotyping

Venous blood (3 mL) was collected from each subject and anticoagulated with EDTA. Genomic DNA was extracted with DNA extraction kit (Wizard Genomic DNA purification kit; Promega, USA). UV spectrophotometer was used to determine the concentration and purity of extracted DNA. The genomic DNA was stored at −20°C.

Four genetic loci of *NOS1* (rs1483757, rs7308402, rs2293050, and rs2139733) were selected according to the gene sequence of *NOS1* in dbSNP database of NCBI (http://www.ncbi.nlm.nih.gov/SNP/). SNaPshot multiple Minisequencing technology was used for genotyping [23].

First, genomic DNA underwent amplification by multiplex PCR with the following mixture (20 *μ*L): 1xGC buffer I, 3.0 mM Mg^2+^, 0.3 mM dNTP, 1 U of HotStarTaq polymerase (Qiagen Inc.), 1 *μ*L of DNA, and 1 *μ*L of multiplex PCR primers. The primers were as follows:

 rs1483757F: 5′-TGCCTCCGACAACTGAGCTGAT-3′
 rs1483757R: 5′-GCCTGCGTGACAGAGTCAAATTC-3′
 rs7308402F: 5′-GCAGGCTTATCCCATGGCTCTT-3′
 rs7308402R: 5′-CCTCTGCTGGGGCATATTTCAA-3′
 rs2293050F: 5′-ATGGCAGACCTGTGGTGGAGAG-3′
 rs2293050R: 5′-CCCTCCACCGTTTTCCTCACAC-3′
 rs2139733F: 5′-GAACACCCTGACCTTAGCTGAC-3′
 rs2139733R: 5′-TTTTGTTGAACCTGGGCCTCTT-3′.

Amplification was done under the following condition: 95°C 2 min; 11 cycles of 94°C for 20 s, 65°C–0.5°C/cycle for 40 s and 72°C for 90 s; 24 cycles of 94°C for 20 s, 59°C for 30 s and 72°C for 90 s; 72°C for 2 min. Then, 10 *μ*L of PCR products was mixed with 1 U SAP (Promega) and 1 U Exonuclease I (Epicentre) followed by incubation at 37°C for 1 h and 75°C for 15 min for inactivation. The PCR products were purified. SNaPshot multiple single-base extension reaction was done with the following mixture (10 *μ*L):  5 *μ*L of SNaPshot Multiplex Kit (ABI), 2 *μ*L of purified products from multiplex PCR, 1 *μ*L of primers for extension, and 2 *μ*L of ultrapure water. The PCR conditions were 96°C for 1 min, 28 cycles of 96°C for 10 s, 52°C for 5 s, and 60°C for 30 s. Then, 10 *μ*L of products from extension was mixed with 1 U SAP followed by incubation at 37°C for 1 h and 75°C for 15 min for inactivation. Then, 0.5 *μ*L of purified products from extension was mixed with 0.5 *μ*L of Liz120 SIZE STANDARD and 9 *μ*L of Hi-Di followed by denaturation at 95°C for 5 min. The products were then subjected to sequencing (ABI3130XL). The raw data were analyzed with GeneMapper 4.0 (AppliedBiosystems Co., Ltd., USA) for genotyping.

### 2.3. Statistical Analysis

Statistical analysis was done with SPSS version 16.0. Data were expressed as mean ± standard deviation (SD) or percentage. Demographics, risk factors, genotype frequency, and allele frequency were compared with Student's *t*-test or Pearson's *x* ^2^. The correlation of gene polymorphism with IS was analyzed with logistic regression analysis after adjusting traditional risk factors of stroke (such as blood pressure, blood lipid, blood glucose, and history of smoking and drinking). The odds ratios (OR) and 95% confidence intervals were calculated before and after adjusting confounding factors. Chi-square goodness-of-fit test was used to test whether SNP genotype frequency met the Hardy-Weinberg equilibrium. Online SHEsis software was employed to analyze the linkage disequilibrium and haplotype of 4 SNPs. A value of *P* < 0.05 was considered statistically significant.

## 3. Results

Clinical information was collected at baseline from all subjects and compared between patients and controls (Table 1). There were no marked differences in the age, gender, body mass index (BMI), and high-density lipoprotein cholesterol (HDL-C) between the two groups. However, marked differences were noted in the history of hypertension, history of diabetes, history of smoking, history of drinking, systolic blood pressure (SBP), diastolic blood pressure (DBP), fasting blood glucose (FBG), total cholesterol (TC), triglyceride (TG), and low-density lipoprotein cholesterol (LDL-C) between the two groups (*P* < 0.05) (Table 1).

The genotype distribution of rs1483757, rs2139733, rs2293050, and rs7308402 met the Hardy-Weinberg equilibrium (*P* > 0.05). In patients, the *P* value was 0.635, 0.760, 0.962, and 0.492, respectively; in controls, the *P* value was 0.162, 0.111, 0.105, and 0.227, respectively. This suggests that this population is representative in North China. The genotypes of SNP sites and allele frequency in patients and controls are shown in Table 2. Results showed that there were no marked differences in the genotypes and allele frequency of rs1483757, rs2139733, and rs2293050 between patients and controls (*P* > 0.05). However, the AG genotype frequency and A allele frequency of rs7308402 in IS patients were markedly lower than those in controls (*P* = 0.037 and *P* = 0.041, resp.). Stratification analysis of females and males revealed that the AG genotype frequency and A allele frequency were different in female patients and female controls (*P* = 0.001 and *P* = 0.002, resp.). Nevertheless, this difference was not observed between male patients and male controls (Table 3). After adjusting confounding factors (such as gender, age, smoking, drinking, hypertension, diabetes, blood lipid, and blood glucose), the AG genotype frequency and A allele frequency were still associated with IS, and both were protective factors (OR = 0.572, 95% CI: 0.335–0.978, *P* = 0.041, and OR = 0.611, 95% CI: 0.378–0.985, *P* = 0.041, resp.) (Table 2). Similar finding was also observed in female patients in stratification analysis (OR = 0.328, 95% CI: 0.153–0.703, *P* = 0.004, and OR = 0.347, 95% CI: 0.174–0.691, *P* = 0.002, resp.) (Table 4).

The online SHEsis software was employed to test the pair linkage disequilibrium of 4 sites. Results showed linkage disequilibrium in rs2139733 and rs2293050 (*P*_1_ = 0.995,  *P*_2_ = 0.977). The frequency of potential haplotypes composed of 4 sites (rs1483757, rs2139733, rs2293050, and rs7308402) was also evaluated in patients and controls with SHEsis. Results showed that GATA haplotype frequency in IS patients was dramatically lower than that in controls, suggesting that GATA haplotype is a protective haplotype (OR = 0.593, 95% CI: 0.361–0.977, *P* = 0.038) (Table 5).

## 4. Discussion

In the present study, 4 SNP sites (rs1483757, rs2139733, rs2293050, and rs7308402) of *NOS1* gene were detected in Han Chinese with IS of North China, and their relation with IS was evaluated in this population. Our results showed that the genotypes and allele frequency of rs1483757, rs2139733, and rs2293050 were comparable between IS patients and control, but the AG genotype and A allele frequency of rs7308402 were markedly reduced in IS patients when compared with healthy controls (*P* = 0.037, *P* = 0.041). After adjusting traditional confounding factors (gender, age, hypertension, diabetes, smoking, drinking, and blood lipid), the AG genotype and A allele frequency of rs7308402 were still related to IS (OR = 0.572, 95% CI: 0.335–0.978, *P* = 0.041 and OR = 0.61, 95% CI: 0.378–0.985, *P* = 0.041, resp.). These findings suggest that SNP of rs7308402 may be a genetic marker of female Han patients with IS in North China. Yamaguchi et al. [24] and our previous studies [25, 26] also revealed the difference in some SNPs related to IS between males and females, which was also confirmed in this study. The cause of this difference is still unclear and the difference in sex hormone might be an attributor. Lekontseva et al. [27] investigated the resistant vessels in female animals. Their results showed nNOS mediated dilation of arteries *in vitro*.

Haplotype analysis may overcome the disadvantage of little information provided by SNP analysis. Haplotype has low recombination rate and high stability. There is evidence showing that haplotype analysis is superior to single SNP analysis, especially when the linkage disequilibrium of SNPs is very weak [28]. Our results showed that GATA (rs1483757-rs2139733-rs2293050-rs7308402) was a susceptibility haplotype. The GATA frequency in IS group was markedly lower than that in controls. Subjects with GATA haplotype had a lower risk for IS (OR = 0.593, 95% CI: 0.361–0.977, *P* = 0.038), suggesting that GATA is a protective haplotype.

Studies on nNOS knockout and nNOS overexpression animals significantly improve our understanding of the pathophysiology of nNOS-derived NO [12]. In animals with diet-induced atherosclerosis, *ApoE*/nNOS knockout mice had more severe atherosclerosis than those with *ApoE* knockout alone [14]. In addition, nNOS expression is also found in atherosclerotic plaques, suggesting that nNOS plays an important role in the endothelial inflammation [29, 30]. Recently, Chakrabarti et al. [31] found nNOS is involved in the NO production in the endothelial cells at rest, and NO may exert anti-inflammatory effect via reducing proinflammatory cytokines. Thus, *nNOS* is also regarded as a novel antiatherosclerotic factor. Our results showed that the *nNOS* gene polymorphism was a protective factor, which was consistent with the antiatherosclerotic property of nNOS. In addition, Nakata et al. [32] found that statins, drugs that can lower cholesterol in hypercholesterolemia patients, could upregulate nNOS expression in human endothelial cells, rat vascular smooth muscle cells, and mouse aorta, suggesting that statins may reduce the risk for stroke in a novel vascular mechanism which is independent of cholesterol-lowering effect of nNOS. There is evidence [9] showing that NO in central nervous system is involved in the central regulation of blood pressure and inhibits nNOS activity in the medulla oblongata and hypothalamus resulting in systemic increase in blood pressure. NO from activated peripheral nitrogen source nerves may dilate peripheral blood vessels, reduce peripheral resistance, and then decrease blood pressure [16]. Seddon et al. [15] found that the activated parasympathetic nerves increased NO production in postganglionic fibers of nitrogen source nerves, which increased blood vessels in the brain and increase cerebral blood flow. Taken together, nNOS is directly related to risk factors of stroke such as atherosclerosis and hypertension. Thus, it is necessary to investigate *nNOS* as a candidate gene of IS.

nNOS protein [21] is composed of PDZ domain, NO synthesis domain, FMN, FAD, and NADH domain. Ca^2+^/calmodulin (CaM) mediated nNOS dimerization and depolymerization is switch of nNOS activation, and PDZ domain mediated protein-protein interaction may precisely regulate this switch temporally and spatially. Our results showed stroke related rs7308402 located in intron 2 and thus had no function. Thus, we speculate that there is linkage disequilibrium in the functional site of adjacent extron 2 which encodes PDZ domain, which influences the nNOS activity.

Our results showed rs7308402 was related to IS and served as a protective factor, which was consistent with results in the study of Manso et al. [22]. However, our findings did not reveal the correlation of rs2293050, rs2139733, and rs1483757 with IS, which was not consistent with findings in the study of Manso et al. [22]. Results in different population might be distinct, which might be attributed to the differences in the genetic background and environmental factors, study design, and statistics. Thus, studies with large sample size are required to confirm the relationship between *NOS1* gene polymorphism and IS.

Taken together, few studies have been conducted to investigate *NOS1* gene polymorphism and IS. Our findings for the first time indicated that AG genotype and A allele at rs7308402 of *NOS1* gene may reduce the risk for IS in Han Chinese of North China, especially in females. Thus, both might be protective factors of IS. However, the sample size of our study is still small, the sites of nNOS are also limited, and the *NOS1* expression is not detected. Thus, studies with large sample size, more haplotypes, and more examinations are required to functionally confirm the relationship between *NOS1* gene and cerebrovascular disease in Han Chinese.

## Figures and Tables

**Table 1:** Clinical information of IS patients and controls at baseline.

Variables	IS patients (*n* = 413)	Controls (*n* = 477)	*P*
Age	63.47 ± 11.57	62.68 ± 6.32	0.196
Gender (M/F)	201/212	224/253	0.638
BMI (kg/m^2^)	24.28 ± 3.85	24.01 ± 3.72	0.422
SBP (mmHg)	143.63 ± 18.35	132.04 ± 14.46	<0.001
DBP (mmHg)	83.87 ± 10.32	78.76 ± 8.82	<0.001
FBG (mmol/L)	6.41 ± 2.60	5.85 ± 1.71	<0.001
TC (mg/dL)	5.10 ± 0.97	4.96 ± 1.08	0.044
TG (mmol/L)	2.02 ± 3.49	1.63 ± 1.16	0.024
HDL-C (mmol/L)	1.31 ± 0.32	1.33 ± 0.21	0.149
LDL-C (mmol/L)	2.95 ± 0.69	2.92 ± 0.62	0.015
Hypertension (*n*/%)	261 (63.5)	174 (36.5)	<0.001
Diabetes (*n*/%)	102 (24.7)	78 (16.4)	0.020
Smoking (*n*/%)	172 (41.6)	101 (21.2)	<0.001
Drinking (*n*/%)	122 (29.5)	63 (13.2)	<0.001

**Table 2:** Genotype and allele frequency of 4 sites of *NOS1* gene^*※*^.

Genotype	Controls (%)	Patients (%)	OR (95% CI)	*P*	Adjusted OR (95% CI)	*P*
rs1483757						
AA	93 (22.5)	88 (18.4)	1.00	—	1.00	—
AG	211 (51.1)	251 (52.6)	0.80 (0.56–1.12)	0.193	0.81 (0.56–1.19)	0.284
GG	109 (26.4)	138 (28.9)	0.75 (0.51–1.10)	0.138	0.74 (0.48–1.12)	0.737
A allele	397 (48.1)	427 (44.8)	1.00	—		
G allele	429 (51.9)	527 (55.2)	1.14 (0.95–1.38)	0.163		
rs2139733						
AA	80 (19.4)	83 (17.4)	1.00	—	1.00	—
AT	207 (50.1)	252 (52.8)	0.85 (0.60–1.22)	0.381	0.87 (0.59–1.29)	0.484
TT	126 (30.5)	142 (29.8)	0.92 (0.62–1.36)	0.677	0.92 (0.60–1.42)	0.714
A allele	367 (81.3)	418 (84.9)	1.00	—		
T allele	459 (18.7)	536 (15.1)	0.99 (0.77–1.29)	0.960		
rs2293050						
CC	130 (31.5)	143 (30.0)	1.00	—	1.00	—
CT	203 (49.2)	252 (52.8)	0.89 (0.66–1.20)	0.431	0.86 (0.64–1.23)	0.885
TT	80 (19.4)	82 (17.2)	1.07 (0.73–1.58)	0.722	1.07 (0.70–1.64)	0.764
C allele	463 (56.1)	538 (56.4)	1.00	—		
T allele	363 (43.9)	416 (43.6)	1.05 (0.81–1.36)	0.734		
rs7308402						
GG	386 (93.5)	427 (89.5)	1.00	—	1.00	—
AG	27 (6.5)	50 (10.5)	0.60 (0.37–0.97)	0.038	0.57 (0.34–0.98)	0.041
AA	—	—	—	—	—	—
G allele	799 (96.7)	904 (94.8)	1.00	—		
A allele	386 (3.3)	434 (5.2)	0.61 (0.38–0.99)	0.041		

^*※*^Adjusting gender, age, hypertension, diabetes, smoking, drinking, serum LDL-C, and blood glucose.

**Table 3:** Genotype and allele frequency in males and females.

	Males			Females		
	Patients (%)	Controls (%)	*P*	Patients (%)	Controls (%)	*P*
rs1483757						
AA	48 (23.9)	45 (20.1)	0.580	45 (21.3)	43 (17)	0.328
AG	99 (49.3)	120 (53.6)		112 (52.8)	131 (51.8)	
GG	54 (26.9)	59 (26.3)		55 (25.9)	79 (31.2)	
A	195 (48.5)	210 (46.9)	0.634	202 (47.6)	217 (42.9)	0.146
G	207 (51.5)	238 (53.1)		222 (52.4)	289 (57.1)	
rs2139733						
AA	41 (20.4)	42 (18.8)	0.902	39 (18.4)	41 (16.2)	0.591
AT	98 (48.8)	110 (49.1)		109 (51.4)	142 (56.1)	
TT	60 (30.8)	72 (32.1)		64 (30.2)	70 (27.7)	
A	180 (44.8)	194 (43.3)	0.573	187 (44.1)	224 (44.3)	0.960
T	222 (55.2)	254 (56.7)		237 (55.9)	282 (55.7)	
rs2293050						
CC	62 (30.8)	73 (32.6)	0.842	68 (32.1)	70 (27.7)	0.364
CT	98 (48.8)	110 (49.1)		105 (49.5)	142 (56.1)	
TT	41 (20.4)	41 (18.3)		39 (18.4)	41 (16.2)	
C	222 (55.2)	256 (57.1)	0.573	241 (56.8)	282 (55.7)	0.734
T	180 (44.8)	194 (42.9)		183 (43.2)	224 (44.3)	
rs7308402						
GG	185 (92)	210 (93.8)	0.492	201 (94.8)	217 (85.8)	0.001
AG	16 (8)	14 (6.2)		11 (5.2)	38 (14.2)	
AA	—	—		—	—	
G	386 (96)	434 (96.9)	0.499	413 (97.4)	470 (92.9)	0.002
A	16 (4)	14 (3.1)		11 (2.6)	36 (7.1)	

**Table 4:** Regression analysis in females^*※*^.

	Adjusted OR	95% CI	*P*
rs1483757			
AA	1	—	—
AG	0.921	(0.533–1.591)	0.768
GG	0.738	(0.405–1.346)	0.322
rs2139733			
AA	1.00	—	—
AT	0.775	(0.441–1.363)	0.484
TT	0.912	(0.490–1.696)	0.770
rs2293050			
CC	1.00	—	—
CT	0.749	(0.466–1.206)	0.234
TT	1.014	(0.547–1.880)	0.964
rs7308402			
GG	1.00	—	—
AG	0.328	(0.153–0.703)	0.004

^*※*^Adjusting gender, age, hypertension, diabetes, smoking, drinking, serum LDL-C, and blood glucose.

**Table 5:** Haplotype frequency of *NOS1* gene in patients and controls.

rs1483757-rs2139733 -rs2293050-rs7308402	Patients (%)	Controls (%)	*P*	OR (95% CI)
A A T G	9.6	9.1	0.714	1.062 (0.771–1.462)
A T C G	38.3	35.6	0.197	1.136 (0.936–1.378)
G A T A	3.0	4.9	0.038	0.593 (0.361–0.977)
G A T G	31.2	29.5	0.408	1.089 (0.889–1.335)
G T C G	16.8	20.3	0.072	0.801 (0.630–1.020)

Note: Haplotypes with frequency of higher than 3% are shown.
